# Bringing the Walk with Ease Programme to the UK: a mixed-methods study to assess the relevance, acceptability, and feasibility of implementation for people with arthritis and musculoskeletal conditions

**DOI:** 10.1093/tbm/ibad032

**Published:** 2023-06-15

**Authors:** Kathryn R Martin, Kevin Stelfox, Gary J Macfarlane, Paul McNamee, Zoe Morrison, Toby O Smith

**Affiliations:** Academic Primary Care, Institute of Applied Health Sciences, School of Medicine, Medical Sciences and Nutrition, University of Aberdeen, Foresterhill, Aberdeen AB25 2ZD, UK; Aberdeen Centre for Arthritis and Musculoskeletal Health (Epidemiology Group), School of Medicine, Medical Sciences and Nutrition, University of Aberdeen, Foresterhill, Aberdeen AB25 2ZD, UK; Aberdeen Centre for Arthritis and Musculoskeletal Health (Epidemiology Group), School of Medicine, Medical Sciences and Nutrition, University of Aberdeen, Foresterhill, Aberdeen AB25 2ZD, UK; Aberdeen Centre for Arthritis and Musculoskeletal Health (Epidemiology Group), School of Medicine, Medical Sciences and Nutrition, University of Aberdeen, Foresterhill, Aberdeen AB25 2ZD, UK; Health Economics Research Unit, Institute of Applied Health Sciences, School of Medicine, Medical Sciences and Nutrition, University of Aberdeen, Foresterhill, Aberdeen AB25 2ZD, UK; Aberdeen Business School, Robert Gordon University, Aberdeen, AB10 7QE, UK; Nuffield Department of Orthopaedics, Rheumatology and Musculoskeletal Sciences, University of Oxford, Oxford OX3 7LD, UK; School of Health Sciences, University of East Anglia, Norwich, NR4 7TJ, UK

**Keywords:** Walk With Ease, Arthritis, Cultural adaptation, Evidence-based, Non-clinical intervention, Community walking programme

## Abstract

Developed in the United States (US), Walk With Ease (WWE) is a popular evidence-based, 6-week community walking programme for adults with arthritis, delivered in either an instructor-led or self-directed format. While WWE has expanded into communities across the USA, it is relatively unknown in other countries across the globe. This study, in collaboration with community and patient partners, aimed to examine the relevance, acceptability and feasibility of introducing WWE into a UK context. After initial cultural adaptation, participants were recruited into the study. Eligible (≥18 years, doctor diagnosed arthritis (confirmed or self-report), self-reported joint symptoms in last 30 days, BMI ≥25 kg/m^2^, and <150 min/week of moderate/vigorous PA) and consented participants were randomized into two groups: WWE programme or usual care. A mixed-methods analysis approach integrated quantitative data (physical performance assessment; baseline and post-six week programme questionnaire) and qualitative data (narrative interviews exploring participants’ pre- and post-WWE experiences and stakeholders’ perceptions). Of 149 participants, the majority were women (70%) aged ≥60 years (76%). Among the 97 receiving the programme, 52 chose instructor-led; 45 chose self-directed. Participants found WWE relevant and acceptable—99% indicating they would recommend WWE to family/friends. Within both WWE formats, mixed differences representing improvement were observed at 6 weeks from baseline for physical performance and arthritis symptoms. Emergent themes included improved motivation, health, and social well-being. WWE is a relevant and acceptable walking programme with scope for wider implementation to support UK health and well-being policy strategies.

## Teaser

Why start from scratch when a successful evidence-based walking programme already exists elsewhere in the world? A culturally adapted WWE programme is acceptable and relevant to a UK population, and can support key UK Government policy initiatives to promote physical activity and social connectedness among adults living with arthritis and musculoskeletal conditions.

**Implications**

**Practice:** Physical activity commissioners, health care providers, and third sector organizations should incorporate Walk With Ease (WWE) UK into programmatic offerings and also as part of social prescribing and physical activity referral schemes.

**Policy:** Policy makers seeking to support UK-wide strategies to tackle physical inactivity, social isolation and loneliness, reduce the burden of arthritis, and musculoskeletal conditions, as well as promote aging initiatives should invest in evidence-based physical activity programming such as WWE-UK.

**Research:** Future research should assess best practice for expansion of WWE within the UK, seek to better understand relevance and acceptability in non-majority communities, and identify barriers to sustainability, particularly among remote and rural populations.

## BACKGROUND

### Importance of physical activity for arthritis and musculoskeletal conditions

Physical inactivity is a major public health challenge. In the UK, it has been estimated that up to 63% of adults are physically inactive [1]. A reduction of 10% in physical inactivity across the primary care population has been estimated to offer considerable health and economic benefits [2]. These include reduced risk of cardiovascular disease, diabetes and some cancers (e.g., breast, colon) [3], and improved control of body weight, mood, and greater health-related quality of life (HRQOL) [3–6].

Physical inactivity is highly prevalent in populations with arthritis and musculoskeletal conditions (MSK). Individuals with osteoarthritis (OA), rheumatoid arthritis (RA), and chronic pain are less active compared to those without [7–10]. However, moderate intensity physical activity can reduce pain, fatigue, stiffness, and disability for people with MSK conditions [11–16]. Despite these known benefits, increasing physical activity is a challenge for individuals with MSK conditions as many are likely to be older, have one or more comorbid health conditions (e.g., diabetes, cardiovascular disease), be fatigued and are fearful of pain exacerbation on activity [17]. There may also be negative health beliefs about their condition which can reduce activity participation [18, 19]. Concerns about navigating community and environmental conditions that may be perceived as unsafe (e.g., uneven pavements) and fear of (re)injury can also adversely impact physical activity levels among those with MSK conditions [20], despite many individuals reporting a desire to become more active [9].

Walking at a moderate/brisk pace (i.e., a metabolic equivalent task value between three and four [21] can help most adults safely achieve recommended physical activity levels). Walking has been reported as acceptable and feasible for people with MSK conditions [22, 23]. It is the preferred form of physical activity for individuals with arthritis [9, 24]. Walking for exercise is associated with significant improvements in pain and self-reported function among individuals with chronic MSK pain [25].

### Walking, arthritis/MSK and place

Although there is considerable evidence to support the promotion of walking [26–28], more needs to be done, especially in primary care settings, to encourage patients with arthritis or MSK conditions to be physically active. Exercise on prescription, referral to physical activity programmes, and/or including physical activity as a self-management strategy as part of a comprehensive care plan may prove successful in promoting behavior change in both the short- and long-term [2, 28, 29]. Scottish Government recommendations suggest “health and care service providers should proactively facilitate walking opportunities within their delivery programmes e.g., GP referral” [26]. These strategies allow a walking intervention to be administered in a non-medicalized community setting with an accessible peer or lay programme leader, rather than requiring more costly health professional input (such as physiotherapists), as has been the case with many previous arthritis or MSK walking programmes [25].

### The Walk With Ease programme

The Arthritis Foundation’s Walk With Ease (WWE) is a 6-week, community-based, walking programme developed in the USA. WWE was specifically designed to affect behavior change for optimal outcome and promote self-efficacy through a participant guidebook [30]. Key motivational strategies in the guidebook include identifying barriers, setting action plans and goals, establishing rewards and identifying and proactively accessing social support and local resources [22]. WWE is offered in separate formats: self-directed or instructor-led. The self-directed format is provided as a guidebook where the participant follows set activities over 6 weeks, including 10- to 30-minute walks, three times weekly, following the “five-step basic walking pattern”: warm-up, gentle stretch, walk, cool-down, gentle stretch. The instructor-led format consists of three, 1-hr group sessions per week that include educational talks and walks following the “five-step basic walking pattern.” Both formats stress that participants should engage with the programme at a comfortable level at the onset, gradually increasing both pace and time spent walking over the 6-week programme. Additionally, participants are encouraged to engage three times a week in strengthening exercises, performed in standing, or seated positions.

### Walk With Ease context

Both formats of WWE have been evaluated in the USA and have been demonstrated to be safe while reducing pain, stiffness and fatigue, improving strength, balance and walking pace, reducing disability and increasing arthritis self-efficacy [22]. Participant satisfaction with both formats has been reported as high [22]. In the USA, the Centers for Disease Control and Prevention (CDC) Arthritis Program has designated WWE as a recognized (group-based) and promising (self-directed) evidence-based physical activity programme for lifestyle management of arthritis [31]. Despite the success of WWE, it has yet to be introduced outside of the USA.

Beyond obvious linguistic variations, there exist cultural differences that can influence arthritis/MSK self-management through uptake of a community-based physical activity ­intervention. Within the UK, the walking culture is perceived to be different to that in the USA. In Scotland, there is a tradition of rambling protected by the Right to Roam [32]. There is less emphasis on personal vehicles due to high petrol and running costs and subsequent greater uptake of public transport due to free bus travel for those over age 60 years across the UK and the under 22s in Scotland. Location of amenities encourages active transport (e.g., nearly 85% of the English population live within a 20-min walk to their GP surgery [33]). The UK also experiences fewer extremes in weather conditions than the USA, and there is a general acceptance that rain or bad weather is always a possibility. Our community and patient partners recognized the potential of WWE to capitalize on this walking culture as there is no widely available evidence-based walking programme in the UK for people with arthritis/MSK. Yet adopting it “as is” was not an option as it might be perceived as redundant given there is already a culture of walking for leisure and active transport within the UK. Overall, we seek to contribute to the awareness that cultural adaptation of socially situated community-based health interventions such as WWE is important and necessary.

The RE-AIM framework [34] guided the development of the wider mixed-methods feasibility study to ensure capture of appropriate information for thorough reflection of the process. The research reported in this paper focusses on the RE-AIM components of reach, effectiveness, and maintenance as it aimed to assess how WWE could be best adapted for a UK context. Future work will report on the RE-AIM components of adoption and implementation, as these are outside the scope of the current paper.

### Aims and objectives

Specifically, this study aimed to examine the relevance, acceptability and feasibility of introducing WWE into a UK context. Our objectives were to:

1. Identify and make cultural adaptations to ensure the relevance and feasibility of adoption of WWE in a UK setting;
2. Examine aspects of recruitment, randomization, assessment compliance, extent of data variation, and adherence to activity to inform the design of a future trial of WWE in the UK;
3. Explore the perceptions and experiences of WWE among participants, community partners and community organizations to identify processes implicit in integrating a community-based walking programme for individuals with arthritis or MSK conditions into current care.

## METHODS

This study used a longitudinal embedded mixed-methods design [35]. The benefit of this approach was that it could both quantitatively investigate changes using a pre- and post-set of physical indicators and to qualitatively describe the perceptions and experiences of WWE among participants and key stakeholders [36].

### Identification of cultural appropriateness and areas for adaptation

We followed a two-step process to identify to what extent WWE was culturally appropriate and the extent to which ­cultural adaptation would need to be undertaken without changing the core fundamental components of the programme. Firstly, research team members (*n* = 4) worked alongside scientific advisors (*n* = 2) and community partners (*n* = 3) to identify and make changes deemed culturally appropriate. Secondly, this was followed by a workshop with patient partners (*n* = 6) to discuss the programme and guidebook. Further suggestions were then incorporated into the materials to update both the participant guidebook and the walk leader manual.

### Recruitment and training of walk leaders

A snowball approach was used for recruiting walk leaders through community partners and a city-wide volunteer organization. Six potential walk leaders attended a 2-hr meeting comprising a comprehensive discussion on arthritis/MSK conditions, the WWE programme, walk leader roles and expectations, and an overview of the study. Four individuals completed a Cardiopulmonary Resuscitation (CPR) certification course and the online WWE training programme run by the Athletics and Fitness Association of America (AFAA) to become a certified WWE instructor. These instructors then lead group walks for this study and received £200 to thank them for their time.

### Testing and evaluation of WWE-UK programme

This study had ethical approval from the South-East Scotland Research Ethics Committee 02 (Reference: REC: 17/SS/0069).

The primary recruitment approach was facilitated through the Scottish Primary Care Research Network (SPCRN). A patient medical records review identified potentially eligible participants from nine primary care practice from around a large city in Scotland using relevant arthritis and MSK conditions clinical codes (e.g., knee/hip OA, RA, gout, fibromyalgia). Lists were reviewed by a General Practitioner (GP) or medical practice manager, which allowed for identification and removal of patients from the list who would not be able to exercise due to unstable cardiovascular status, cognitive issues, or complex medical conditions. Remaining individuals were invited by letter. A secondary recruitment strategy used targeted advertising through relevant community organizations (e.g., University of Aberdeen, local community centers and libraries, NHS Grampian Public Involvement, Aberdeen and Grampian Chamber of Commerce, and national/local charities) to encourage interested members of the public to self-refer to the study.

All eligible participants were required to meet all the inclusion criteria:

1. Aged 18 years or older;
2. Have arthritis or a MSK condition (e.g., OA, RA, back pain, gout or fibromyalgia), either by a confirmed diagnosis in medical notes or self-reported by participant diagnosis by a clinician;
3. Have self-reported symptoms of pain, aching or stiffness in and/or around a joint (including the lower back) in the previous 30 days;
4. Self-reported height and weight indicating being overweight or obese (body mass index ≥25 kg/m^2^);
5. Self-reported current physical inactivity (i.e., less than 150 min total moderate-to-vigorous physical activity per week on average).

All eligible participants were invited to a baseline study visit, where they were asked to provide consent to participate in the study. They underwent physical performance measures (PPM) and completed a questionnaire.

At the end of the baseline visit, participants were randomized by use of opaque sealed envelopes to the WWE-UK walking programme or usual care group in a 2:1 ratio respectively. Those allocated to the WWE-UK walking programme were offered the choice of walking either in: (a) an instructor-led group or (b) via self-directed walking. They were given a WWE-UK guidebook and a pedometer. Those allocated to the usual care group were provided with a leaflet about opportunities to be active in the local community, for example, locations and types of physical activity programmes offered by six city-based organizations.

All participants, regardless of study group, were invited to return for a repeat physical performance tests and follow-up questionnaire after the 6-week WWE programme and to complete a mailed questionnaire 18-weeks post-baseline assessment. A list of specific data collected and instruments used in the questionnaires, along with individual references, is presented in Supplementary Material File 1.

We embedded a qualitative stage to better understand perceptions of the WWE programme from various viewpoints. Data generation included ethnographic observation of group-led activities during the 6-week WWE programme (not reported here) and narrative interviews with participants post-programme. Semi-structured interviews were also conducted at the end of the programme with walk leaders, community partners and referring physicians (Supplementary Material File 2).

Study participants indicating interest to participate in a one-to-one narrative interview at baseline consent were contacted after the 6-week programme. A sample of 60 participants were recruited to achieve maximum variation by gender, age and programme status (i.e., group, self-directed, or usual care). All the interviews conducted were face-to-face using a pre-tested topic guide (Supplementary Material File 3) by experienced qualitative researchers. All interviews were audio-recorded with the participants’ consent. Researchers took reflective field notes. Audio recordings were professionally transcribed verbatim by a University-approved transcription service and anonymized to maintain confidentiality during analysis.

#### Analysis

Quantitative data: including physical performance assessment and questionnaire data were analyzed using STATA (Version 14, Stata Corp, Texas, USA) to produce descriptive statistics to explore physical performance and arthritis-related symptoms and beliefs collected at baseline, follow-up and 18-weeks. Satisfaction with the WWE programme, both, overall and with specific components (e.g., length, topics) were asked at follow-up and 18-weeks.

Qualitative data: including participant and stakeholder interviews, were stored and analyzed by the researchers using NVivo Software (V12, QSR International Pty Ltd). Interview transcripts were imported into the software as individual cases to allow each participant to be identified by predetermined attributes such as group membership.

An interpretive approach to understand the meanings of participants’ accounts was taken. Transparency in the ­process of data analysis provides a robust approach and allows for possible replication of the findings [37]. An iterative process of deductive thematic analysis was undertaken based on prior factors to initially explore the interview data together with inductive coding of further themes identified from the data.

Integration of qualitative and quantitative data was undertaken, with data presented in a joint analysis display table (Table 1).

**Table 1:** Joint analysis display of qualitative and quantitative data convergence and corroboration

RE-AIM framework component and related theme	Qualitative data	Quantitative data	Data ­convergence	Corroboration
Reach				
Participant Recruitment, including motivating factors for study participation	Narrative interviews (Section 3.2.2)	Recruitment numbers (Figure 1; Section 3.2.1)	Enhance	Narrative interviews provided context for response rate and eligibility rate
Retention, including participant engagement with programme and logistics related to engagement and retention	Narrative interviews (Section 3.3.1)	Loss-to-follow-up (Figure 1; Section 3.3.1)	Enhance	Narrative interviews provided insight into participant barriers and motivators to remain engaged with programme and research study
Effectiveness^*^				
Arthritis/MSK symptoms and physical functioning	Narrative interviews (Section 3.4.1)	Questionnaire data (Table 4)	Mixed	Data did not fully converge across all outcomes
General health and other medical conditions	Narrative interviews (Section 3.4.2)	Questionnaire data (Section 3.4.2)	Enhance	Where data absent on other chronic health symptoms, narrative interviews provided context
Confidence and self-efficacy	Narrative interviews (Section 3.3.3)	Questionnaire data (Table 4)	Mixed	Quant data did not fully reflect narrative reports
Life satisfaction and well-being	Narrative interviews (Section 3.3.4)	Questionnaire data (Table 4)	Mixed	Quant data did not fully reflect narrative reports
Maintenance				
WWE-UK Programme Satisfaction	Narrative interview (Section 3.5.1)	Questionnaire data (Section 3.5.1)	Confirm	Data fully converged
Use of Programme Literature/Guidebook	Narrative interviews (Section 3.5.2)	Questionnaire data (Section 3.5.2)	Mixed	Data did not converge as quant data indicated engagement with materials, but narrative interviews identified barriers to maintenance use
Walking behavior	Narrative and stakeholder interviews (Section 3.4.4)	Questionnaire data (Section 3.5.3)	Confirm	Data indicated participants maintained walking

^*^Study was underpowered to demonstrate effectiveness.

## RESULTS

### Cultural adaptation of the WWE guidebook/manual

Overall, there was good agreement between stakeholders indicating that guidebook content was appropriate for a UK context. The programme was thought to be relevant and well-presented, with praise given for the positive way information was presented, elements of self-management advice, activities, and exercises. Research team members and advisors identified a number of language elements within the guidebook that needed tailoring from a USA to a UK audience (sidewalk to pavement; Tylenol to paracetamol).

Further, there were also practical suggestions that included updating formatting (e.g., font, choice of contrasted print colors) and printing-options (e.g., choice of book size—A4 and A5 options). Patient partners requested the guidebook be spiral bound for ease of holding for people with hand/dexterity issues which. Finally, the resources and references section were also adapted to include resources from Versus Arthritis and other UK arthritis/pain charity organizations (e.g., National Rheumatoid Arthritis Society, Pain Concern). The Walk Leader’s Guide was also tailored in a similar manner.

### Reach

#### Participant recruitment

**Fig 1:**
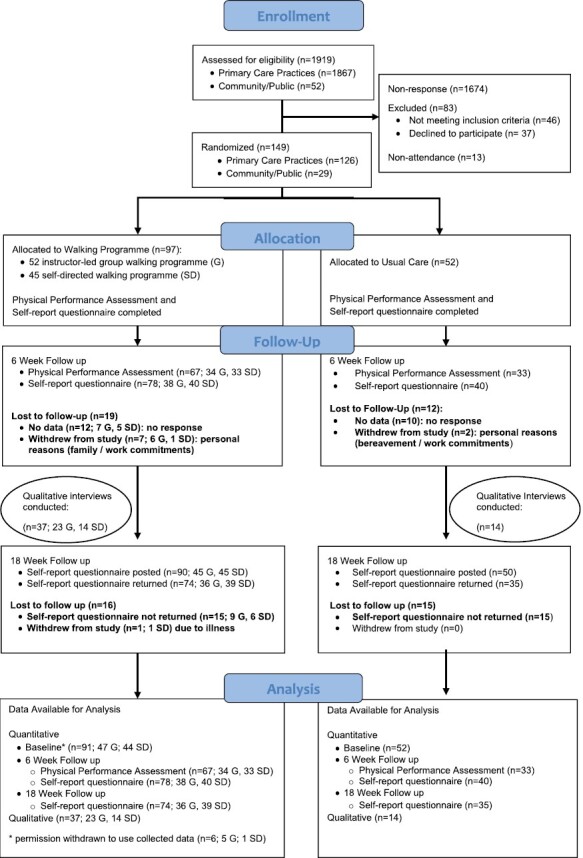
Participant recruitment flow diagram.

From a list of 2,250 potential patients, 1,867 (83%) were posted a study information leaflet and General Practitioner (GP) letter following GP review. There were 193 responses to the posted information with the majority (*n* = 126;65.3%) of respondents were eligible to take part in the WWE-UK study. These figures represent an overall response rate of 10.3%, with an overall eligibility rate of 6.7% for this recruitment approach (Fig. 1). The secondary recruitment strategy resulted in 52 members of the public contacting the team. Of these, five (10%) did not return communication with the research team, 11 (21%) did not meet the inclusion criteria, and 36 (69%) were eligible, with 34 (65%) attending the first baseline visit.

In total, 149 participants attended the first study visit, gave consent and were then randomized. From these, 97 (65%) were allocated to the WWE programme (52 chose the instructor-led option and 45 the self-directed option) and 52 (35%) were randomized to the usual care group. Participants were mostly women (70.5%), of older age [mean 64 years (range 25–86 years)], currently married or partnered (61%), with a mean BMI of 33.4 kg/m^2^ (range 24.7–62.4). The most frequently reported arthritis and MSK conditions were osteoarthritis (OA), back pain, rheumatoid arthritis (RA) and fibromyalgia (Table 2).

**Table 2:** Baseline characteristics by group allocation of all study participants

Baseline characteristics	Walking programme		Usual care
	Instructor-led (*n* = 47)	Self-directed (*n* = 44)	Activity leaflet (*n* = 52)
Demographics			
Age, mean ± *SD* years	65 ± 10	63 ± 12	65 ± 11
Female, %	70.2%	70.5%	67.3%
BMI ≥30 kg/m^2^, %	72.3%	65.9%	63.5%
Arthritis/MSK condition, % yes			
Osteoarthritis	57.5%	61.4%	76.9%
Back pain	44.7%	59.1%	57.7%
Rheumatoid arthritis	19.2%	6.8%	15.4%
Fibromyalgia	10.6%	13.6%	9.6%
Smoking			
Current	17.0%	6.8%	1.9%
Ex-smoker	34.0%	47.7%	48.1%
Never smoker	49.0%	45.5%	50.0%
Performance-based physical function, mean ± *SD* s			
Lower extremity, chair stand^*^	19.85 ± 8.76	15.50 ± 5.65	17.77 ± 8.67
Turning ability, 360° turn	4.01 ± 1.78	3.55 ± 2.20	3.43 ± 1.22
Balance, single leg stance	9.91 ± 12.36	18.21 ± 21.98	17.75 ± 21.26
Walking speed, *normal*	2.78 ± 1.13	2.41 ± 0.92	2.55 ± 0.83
Aerobic endurance, 2 min step test	52.7 ± 23.60	53.18 ± 19.93	57.04 ± 23.52
Arthritis symptoms			
Pain, NRS (range 0–10)	5.40 ± 2.73	5.93 ± 2.33	5.35 ± 2.50
Fatigue, NRS (range 0–10)	5.34 ± 3.09	4.89 ± 2.78	4.73 ± 2.90
Stiffness, NRS (range 0–10)	5.70 ± 2.56	5.89 ± 2.42	5.65 ± 2.42
Psychosocial			
Arthritis self-efficacy—pain (range 1–10)	5.6 ± 2.40	4.99 ± 1.59	5.32 ± 2.09
Arthritis self-efficacy–symptom ASE (range 1–10)	5.77 ± 2.34	5.85 ± 1.82	6.24 ± 1.98
Rheumatology Attitudes Index (RAI) (range 1–5)	2.95 ± 0.90	2.65 ± 0.78	2.60 ± 0.76
Self-efficacy for physical activity (SEPA) (range 1–7)	4.21 ± 1.53	4.04 ± 1.43	4.07 ± 1.44
Outcome expectations for exercise (range 1–5)	2.32 ± 0.81	2.32 ± 0.51	2.24 ± 0.59
Social desirability (range 0–10)	6.19 ± 1.98	6.82 ± 1.93	6.63 ± 1.55

^*^ *n* varies due to missing data [47 (instructor-led); 43 (self-directed); 50 (usual care)].

Of the initial 60 participants invited for a narrative interview, nine did not complete this component due to illness and/or other personal reasons. In total, 51 participants took part in a face-to-face interview which lasted, on average, 60 min. They were generally representative of the wider study, as participants were mostly women (61%), of older age [mean 65 (range 28–86), currently married or partnered (63%), with a mean BMI of 32.5 kg/m^2^ (range 25.1–62.4)] (Table 3). The views of 14 key stakeholders including, walk leaders, professionals from Aberdeen City Health and Social Care Partnership, Sport Aberdeen, Paths for All, and primary care physicians were also captured in semi-structured interviews.

**Table 3:** Baseline characteristics by group allocation of narrative interview participants

Baseline characteristics	Walking programme		Usual care
	Instructor-led (*n* = 23)	Self-directed (*n* = 16)	Activity leaflet (*n* = 12)
Demographics			
Age, mean ± *SD* years	66 ± 8	64 ± 13	67 ± 14
Female, %	52.2%	62.5%	75.0%
BMI ≥30 kg/m^2^, %	73.9%	56.3%	41.7%
Arthritis/MSK condition, % yes			
Osteoarthritis	60.9%	81.3%	66.7%
Back pain	39.1%	62.5%	83.3%
Rheumatoid arthritis	17.4%	6.3%	16.7%
Fibromyalgia	4.4%	18.8%	8.3%
Smoking			
Current	21.8%	–	–
Ex-smoker	39.1%	56.3%	50.0%
Never smoker	39.1%	43.7%	50.0%

#### Motivating factors for study participation

Most participants reported being physically active prior to the onset of their condition and attributed their reduced activity to their MSK condition. This was reflected in a theme identified from the interview data in which individuals had ceased or reduced physical activities such as, golf, walking, swimming and dancing and everyday activities such as, shopping and housework. There was a sense of “loss” as participants felt they had been limited by their condition. In some cases, this had impacted on their general sense of wellbeing.

*I’m not coping very well with it actually because I used to love to go swimming and I used to do a lot of walking, now I can’t, and it’s getting me down.* P11: Male, aged 75 (Instructor-led)

There was evidence from the interview data that a significant motivating factor for engaging with the programme was through the referral route, that is, a letter from their GP.

Earlier on this year my GP sent me a letter asking me if I… inviting me to join the Walk with Ease study. So I took the opportunity to give it a try; nothing ventured, nothing gained. P17: Male, aged 68 (Self-directed)

### Retention

#### Participant engagement with the WWE-UK programme

Nine participants withdrew from the study (6%; six instructor-led; one self-directed; two usual care) within the first 6-weeks. Personal reasons, not related to the programme content included: poor health/illness, other commitments, change in circumstances and bereavement. Total follow-up/participation rate in physical performance assessment and questionnaire was 79% at 6-weeks (80% WWE programme; 77% usual care) and 73% for the questionnaire at 18-weeks (76% WWE programme; 67% usual care).

Across the five walking groups, participants attended on average 11.5 of the 18 total walks (63%). Once people had been recruited to the study there was a personal commitment to walk regularly and complete the programme. This was particularly the case for those participating in the group walk, for example

*In a group, I’m going to let the group down if I don’t go. So, we encourage each other.* P34: Male, aged 68 (Instructor-led)

Interestingly, participants’ medical condition(s) did not appear to be a barrier to engaging with the programme and associated activities, rather participants noted other challenges and barriers such as lack of time, childcare, summer holidays and appointments.

#### Logistics related to engagement and retention

##### Weather

The climate in this region is highly seasonal, with the average high/low temperatures ranging from 19°C/11°C in July to 9°C/3°C in November (the months the study ran the programme), with a high likelihood of rain and very occasional snow. While weather did not appear to be a barrier to either recruiting or retaining participants in this study, several participants noted that they believed the weather could be a barrier to walk attendance during the winter. Some participants identified potential weather-related barriers, such as slippery surfaces and cold damp weather adversely affecting their medical condition. Some participants reported that their own walking might be reduced during the winter months due to the shorter days and the bad weather.

##### Location

The location of the walks did not appear to be problematic for most participants in terms of accessibility. Most instructor-led walks were located close to the city-center and used community facilities as a meeting point. There was one instructor-led walking group that met directly in the city-center, with walks taking place in and around a large shopping center with good transportation links (bus, train) and a carpark. A few participants from this group commented on being self-conscious about undertaking warm-up/down activities in a city-center location and noted the unappealing nature of traffic noise and pollution, but average walk attendance for this group was high (13/18; 72%).

##### Mixed-ability groups

There were a wide range of walking abilities within each of the instructor-led walking groups. Both participants and leaders reported occasional problems keeping groups together during the walk and arriving back at the same time to ­complete the warm-down exercises. A number of groups made adaptions to the programme to accommodate the different walking abilities, for example, having shorter routes for slower walkers, slower walkers walking together on a loop which were in-line with guidance provided in the walk leader manual. Mixed-ability groups were seen as positive, as they encouraged slower walkers to improve their pace and buoyed self-esteem for some of the faster walkers.

##### Walk leader effectiveness

A key element identified by participants in the instructor-led groups was the role of the walk leaders. They highlighted the provision of general support for group participants in terms of providing conversation, encouragement, affirmation/permission for walking within the context of the WWE programme.

##### Programme pace, length, and structure

The pace of the programme over the 6 weeks was appreciated and was linked to enhanced engagement with WWE by building confidence through the gradual increase in distance each week.

But no, I’d recommend the Walk With Ease to anybody who’s the least little bit, how would I describe it, wants to do a thing but are frightened to overdo it. Right, because Walk With Ease was just nice and slow for a start building you up, and to me it was excellent for anybody that’s got conditions like what I have, excellent. P11: Male, age 75 (Instructor-led)

The programme length (i.e., 6-weeks) was also acceptable to both leaders and participants.

The whole logistics of the programme, I think, was about right. I think six weeks is about right because they get to a stage after six weeks where they can think about doing things independently. Stakeholder 2

In general, the majority of participants deemed the WWE programme structure to be useful. The programme guidebook acted as a key facilitator for setting aims, objectives, and goals. Although the self-directed programme provided less structure in the form of attending a walking group at a set time, the self-directed cohort liked the flexibility of when and where to walk and found that structure was provided through the programme literature. For example, the idea of measuring and recording, that is, goal setting within the programme structure, impacted upon the participant’s activity during the programme and beyond.

*I think what spurred me on was doing this and recording my steps every day, which have gone up from 1,500 roughly to roughly 4,500/5,000, sometimes more depending, yeah. P10: Female, aged 75 (Self-directed)*

Although participants were issued with a pedometer as part of the programme, several participants used mobile phones or other devices to measure their steps throughout the programme. It was reported as being a motivational factor keeping up with both the instructor-led and the self-directed participants within the 6-week programme.

### Effectiveness

#### Arthritis/MSK symptoms and physical functioning

Within both WWE-UK formats, greater differences representing signals of improvement were observed at 6 weeks from baseline, compared to those in usual care, for the majority of physical performance measurements and symptoms (Table 4). This was supported by interview data, as a number of participants discussed their arthritis/MSK specific symptoms, particularly their experience of a marked reduction in pain:

**Table 4:** Change in outcome measures by group allocation

Outcome measures	Baseline Mean (SD)	Baseline—6-week Δ (95% CI)	*n*	Baseline—18-week Δ (95% CI)	*n*
Physical performance measures					
Lower extremity strength—chair stand, s					
Instructor-led	17.89 (7.02)	−3.36 (−1.74, −4.98)	31	–	–
Self-directed	14.65 (4.67)	−1.48 (−0.57, −2.59)	32	–	–
Usual care	17.57 (9.24)	−3.26 (−0.73, −5.78)	30	–	–
360° turn ability, s					
Instructor-led	3.70 (1.73)	−0.44 (−0.98, 0.10)	33	–	–
Self-directed	3.27 (1.80)	−0.33 (−0.64, −0.03)	34	–	–
Usual care	3.57 (1.34)	−0.18 (−0.62, 0.26)	33	–	–
Single leg stance (balance), s					
Instructor-led	10.88 (13.63)	3.21 (−0.19, 6.61)	29	–	–
Self-directed	17.39 (19.37)	1.19 (−3.75, 6.12)	34	–	–
Usual care	17.20 (18.01)	−3.38 (−10.93, 4.16)	33	–	–
Walking speed (functional mobility), m/s					
Instructor-led	0.80 (0.28)	0.09 (0.01, 0.16)	33	–	–
Self-directed	0.87 (0.23)	0.06 (−0.02, 0.15)	34	–	–
Usual care	0.77 (0.22)	0.06 (−0.02, 0.14)	33	–	–
Aerobic endurance—2-min step test, count					
Instructor-led	56.29 (25.79)	8.32 (0.10, 16.55)	31	–	–
Self-directed	54.42 (19.22)	7.09 (1.24, 12.95)	33	–	–
Usual care	57.88 (24.13)	4.94 (−5.30, 15.18)	33	–	–
Arthritis symptoms					
Pain, Visual Numeric Rating Scale (0–10)					
Instructor-led	4.94 (2.63)	−0.67 (−1.29, −0.48)		−0.48 (−1.21, 0.24)	33
Self-directed	5.83 (2.47)	−0.81 (−1.44, −0.18)		−0.72 (−1.45, 0.01)	36
Usual care	5.26 (2.72)	−0.56 (−1.23, 0.11)		−0.56 (−1.30, 0.19)	34
Fatigue, Visual Numeric Rating Scale (0–10)					
Instructor-led	4.64 (3.20)	−1.09 (−1.81, −0.37)		−0.70 (−1.49, 0.10)	33
Self-directed	4.61 (2.73)	−0.47 (−1.20, 0.26)		−0.28 (−0.89, 0.33)	36
Usual care	4.62 (3.02)	−0.65 (−1.54, 0.24)		−0.71 (−1.55, 0.13)	34
Stiffness, Visual Numeric Rating Scale (0–10)					
Instructor-led	5.39 (2.54)	−1.12 (−1.92, −0.32)		−0.73 (−1.41, −0.04)	33
Self-directed	5.64 (2.53)	−0.69 (−1.43, 0.47)		−0.61 (−1.23, 0.01)	36
Usual care	5.21 (2.67)	−0.65 (−1.34, 0.50)		−0.09 (−0.73, 0.56)	34
Psychosocial impact					
Well-being (ICEpop CAPability measure for adults)					
Instructor-led	0.68 (0.18)	0.05 (−0.01, 0.10)		−0.30 (−0.36, 0.24)	33
Self-directed	0.75 (0.19)	0.04 (0.01, 0.07)		−0.03 (−0.09, 0.03)	36
Usual care	0.71 (0.20)	0.02 (−0.01, 0.05)		0.01 (−0.03, 0.05)	34
Health-Related Quality of Life (EQ-5D-5L)					
Instructor-led	0.66 (0.16)	0.05 (0.01, 0.09)		−0.01 (−0.05, 0.03)	33
Self-directed	0.64 (0.18)	0.05 (0.01, 0.08)		0.03 (−0.04, 0.09)	34
Usual care	0.65 (0.19)	0.01 (−0.02, 0.04)		−0.05 (−0.10, −0.01)	36
Global life satisfaction—1 (completely dissatisfied) to7 (completely satisfied)					
Instructor-led	4.97 (1.58)	0.55 (0.03, 1.07)		0.39 (0.04, 0.74)	31
Self-directed	5.34 (1.26)	0.17 (−0.19, 0.54)		0.00 (−0.45, 0.45)	35
Usual care	5.50 (1.57)	−0.05 (−0.47, 0.36)		−0.19 (−0.69, 0.32)	29
Arthritis self-efficacy (ASE)—pain (range 1–10)					
Instructor-led	6.05 (2.42)	0.14 (−0.71, 0.99)		0.44 (−0.51, 1.40)	35
Self-directed	5.14 (1.62)	0.68 (−0.02, 1.37)		0.42 (−0.32, 1.15)	36
Usual care	5.59 (2.12)	0.70 (−0.08, 1.48)		0.56 (−0.27, 1.40)	34
Arthritis self-efficacy (ASE)—symptom (range 1–10)					
Instructor-led	6.27 (2.21)	0.56 (−0.17, 1.30)		0.53 (−0.23, 1.29)	34
** ** Self-directed	5.99 (1.79)	0.59 (−0.07, 1.26)		0.82 (0.31, 1.32)	37
Usual care	6.44 (1.93)	0.20 (−0.33, 0.73)		−0.24 (−0.82, 0.34)	34
Rheumatology Attitudes Index (RAI) helplessness (range 1–5)					
Instructor-led	2.75 (0.92)	−0.27 (−0.46, −0.79)		−0.21 (−0.40, −0.17)	34
Self-directed	2.51 (0.70)	−0.23 (−0.41, −0.60)		−0.11 (−0.32, 0.10)	36
Usual care	2.55 (0.81)	−0.19 (−0.41, 0.03)		0.01 (−0.18, 0.19)	34
Self-efficacy for physical activity (SEPA) (range 1–7)					
Instructor-led	3.23 (1.08)	0.09 (−0.27, 0.45)		0.03 (−0.25, 0.32)	33
Self-directed	2.89 (1.02)	0.27 (0.16, 0.52)		−0.01 (−0.23, 0.22)	36
Usual care	2.83 (1.03)	0.17 (−0.11, 0.45)		0.08 (−0.31, 0.48)	34
Outcome expectations for exercise (range 1–5)^*^					
Instructor-led	2.27 (0.83)	−0.32 (−0.57, −0.07)		−0.20 (−0.38, −0.01)	34
Self-directed	2.34 (0.50)	−0.25 (−0.39, −0.10)		−0.15 (−0.29, −0.12)	36
Usual care	2.16 (0.62)	−0.07 (−0.23, 0.09)		0.01 (−0.15, 0.17)	34

*CI* confidence intervals; *SD* standard deviation; *VNRS* visual numerical rating scale.

^*^1 represents greater outcome expectations.

one thing I noticed was the last week as I say, the knee pain was decreasing. P19: Male, age 72 (Self-directed)

I just think it makes you more flexible, and the pain is not so bad, for myself, pain is not as bad in the evening. P27: Female, age 67 (Instructor-led)

Other participants, from both walking formats, indicated a sense of “feeling good” from completing the activities in the programme. This was put in the context of benefit regardless of major improvements in pain:

what it did for me was re-motivate me, give me a lesson, in actual fact what I knew anyway, if you believe you can do something you can, just do it. So again, I think for me it was more of an emotional thing and getting rid of that malaise. P26: Female, age 57 (Instructor-led)

#### General health and other medical conditions

The majority of participants self-rated their health as good, very good or excellent at baseline—instructor-led: 70.6% (*n* = 34) and self-directed: 69.4% (*n* = 36). A greater number of individuals reported better self-rated health after programme completion (79.4% instructor-led and 75.0% in self-directed). However at 18-weeks, more instructor-led participants maintained the improvement (73.5%) and self-directed returned to baseline (69.4%).

Although questionnaire data did not assess physical health in relation to other chronic medical conditions, participants noted observable improvement in their health during interview. For example, one noted an improvement in respiratory function and another in hypertension:

It’s certainly impacted on fitness…. I do have asthma to a certain extent, and I have a preventative inhaler that I use every day, but the Ventolin, I think it’s called for any attack at the time, I’ve very rarely used it since I’ve been doing the walking scenario. So, that’s a big benefit. P29: Male, age 54 (Instructor-led)

the doctor wanted to get me walking to … to lower my blood pressure, and maybe get rid of some weight. That was the main thing for me. I have a wee blood pressure thing at home and I find that it was certainly taking my blood pressure down, because I think that is what started it. P8: Female, age 75 (Self-directed)

#### Confidence and self-efficacy

A number of participants also expressed “feeling good” about taking part in a physical activity programme, achieving targets, and personal goals. The latter two elements are key features of the programme and identified as facilitators of within-programme engagement by participants. Participants who self-selected into an instructor-led group highlighted a sense of satisfaction that came from being a member of a group, including sense of accomplishment, empowerment and motivation.

As the weeks progressed, I started to do more. I started to pick the pace up a bit. Still not fast but I remember one day walking with the other gentleman and we overtook two people and I haven’t overtaken anybody in a long time. That was a baby step, that was a milestone for me. It’s not much to a lot of people but overtaking someone was brilliant. P3: Male, age 58 (Instructor-led)

While questionnaire evidence did not strongly demonstrate improvements in self-efficacy (arthritis pain, symptoms, or physical activity), data suggest reduced levels of perceived helplessness and enhanced outcome expectations for exercise (Table 4). This differed from themes within the narrative interviews. For example, participants highlighted new appreciation for engaging in appropriate activities and stretching exercises:

I think the main thing it gave to me was a bit of empowerment because it told me how I should be exercising properly. I already had exercises for strengthening my muscles, but it told me the best way to exercise for going on a walk. I think also the fact it told me that I can’t hurt myself walking, was great, because up until then I was worried that I’d get older and older, and sorer and sorer. So, I no longer feel it’s necessarily the case. P23: Male, age 64 (Instructor-led)

There was a general awareness of and increased desire to address health, improved self-care, and help-seeking behavior through changes in daily routine or lifestyle activities through participating in the programme. Several participants reported that they had been encouraged to increase their activity levels in their everyday life.

That’s what I learnt, to be more active. Leave the car a bit away and have a short walk. It doesn’t need to be a long walk. P27: Female, age 67 (Instructor-led)

Others had reframed their belief in their ability to be active and started to undertake other types of physical activity.

The fact that it gave me … I felt a bit more confident about doing stuff, which resulted in going along because I knew there was the over 55 badminton.

P51: Male, age 64 (Instructor-led)

*…. as an outcome of that I now go there regularly; I’m down there three times a week and I’m using the swimming pool and all sorts of things, and that was kick started by this [the WWE-UK Programme]. And as a result of that I’m feeling so much better.*

P26: Female, age 61 (Instructor-led)

#### Life satisfaction and well-being

Benefits gained from being in a group were linked to an enhanced sense of well-being through providing companionship, chatting, having a laugh and being with people who share similar conditions. Participation in the group-led format provided motivational factors, that is, to attend sessions and complete the activities. Post-programme, a modest improvement in global life satisfaction was reported by instructor-led participants in the 6-week questionnaire which was maintained, although reduced at 18-weeks (Table 4).

Similarly, walk leaders and community partners felt the programme had impacted on participants’ health and provided social benefits.

I would say the social impact I think is the biggest thing that I see… is that it just seems to be a really friendly way of getting active but also meeting other people and I’ve heard of people that have gone on walking programmes that have then met people that then go on to do other things. (Stakeholder 6)

In several instances the “group” enhanced the participants’ social networks, some of which were sustained after the programme ended in the form of self-organized social activity networks. One group organized themselves, continued to meet monthly at different area locations and even participated in a parade celebrating city organizations and groups.

### Maintenance

#### WWE-UK programme satisfaction

In general, participants who were interviewed, particularly those in the instructor-led group, spoke of their overall enjoyment with and satisfaction with the programme.

*…I was actually quite negative going into the Walk With Ease programme, I really was. I felt that nothing would work…no matter how much I try, nothing is going to work….*

*But how wrong was I? I enjoyed the programme, I was relaxed, I felt made at ease by the walking co-ordinator…And it was very good.* P03: Male, age 58 (Instructor-led)

At 18-weeks, three-months post-participation, 99% of participants randomized to WWE reported in the questionnaire that they would recommend the programme to their family or friends. The majority (81%) reported they were satisfied with the programme overall (instructor-led: 86%; ­self-directed 77%). This included high satisfaction with programme length, (75%; instructor-led: 75%; self-directed 74%) and programme topics (75%). In addition, participants reported being confident or very confident that the WWE programme increased their knowledge about walking in a safe and comfortable manner (instructor-led: 81%; self-directed: 82%).

#### Use of programme literature/guidebook

Respondents were queried about which sections of the WWE guidebook they continued using since the programme ended. There was some evidence from the post-programme interviews that participants used the programme literature.

I use it [programme and guidebook] regularly. As I say, I keep track of the steps, and it’s all tucked into the guidebook, so the exercises, the repetitiveness of the walking, I am using that all the time, so it’s certainly helping me. It’s very helpful. I believe without it, I wouldn’t do it. So, it’s concentrating me. P29: Male, age 54 (Instructor-led)

In total, 23% (*n* = 13) indicated in the 18-week questionnaire that they did not use any sections, however 77% reported engaging with at least one section (range 1–5). Stretching exercises (65%) and warm-up and cool-down exercises (61%) were the most frequently reported sections used, whereas the walking diary (16%) and walking contract (12%) were the least frequently reported sections used at 18-weeks.

Programme literature received mixed reviews from the participants and stakeholders. On balance, participants believed that it had useful information, and several indicated they had referred back to the guidebook from time-to-time after programme completion. However, some participants thought that the guidebook was too long, had too much detail and queried whether it could be reviewed and either reduced in length or have added signposting (e.g., section tabs).

Well if you could condense it into what I call a booklet, not a novel, it is quite comprehensive, it covers everything, but do you need to cover everything or can you split it up into different areas that you could do it with? P21: Male, age 68 (Instructor-led)

Regarding length, several participants mentioned concern for individuals with learning disabilities, lower literacy and/or cognitive issues. For example, one stated:

*…I think it’s probably one of my worries with just a book, for the last 20 years of working life, I have dealt with people who quite often have learning difficulties, and a lot of people with dyslexia… I think that would be my worry with just a book because quite a lot of people, they are reading, but they are not really taking in. They are maybe not taking in what they are doing, and I think that is why it was nice to have that bit of facilitation.* P51: Male, age 64 (Instructor-led)

And another mentioned, regarding the guidebook,

*I wondered whether that might be slightly off-putting to some participants, particularly if people may be, depending on their literacy levels, or people who had English as a second language or something like that, I wondered whether it might just be a bit too much information.* Stakeholder 7

Others suggested that the information could be reduced and provided in various formats, from a compact booklet that could be carried when walking, to an e-book or app, or audio-recordings.

#### Walking behavior

As highlighted earlier, participants and key stakeholders noted in interview that walking for physical activity was being maintained post-programme. Indeed, upon completion of the WWE programme, 94% (*n* = 72) of participants reported that they had plans to keep walking. At 18-weeks, 76% (*n* = 56) reported by questionnaire that they had continued to walk after programme completion. The majority reported that the length, frequency and distance of their walks had stayed about the same or increased—86% (*n* = 48), 73% (*n* = 41), and 79% (*n* = 44) respectively. On average, these individuals reported walking four days per week (range: 1–7) for nearly an hour (55 min, range 15–180 min). Walking alone was the most frequently reported at 63.6% (*n* = 35), with 27% reported walking with one other person (*n* = 15) and 9% reporting walking with more than one person (*n* = 5). Ten individuals who took part in the instructor-led format reported that they continued to walk with one or more members of their group.

The most frequently [44% (*n* = 8)] reported reason for not continuing to walk for exercise since the WWE programme ended was arthritis symptoms (pain, fatigue, or stiffness), followed by other health problems (including surgery) 33% (*n* = 6), family and life events (22%, *n* = 4), and not having enough time (22%, *n* = 4). However, the majority (83%) of those not continuing to walk did report that they were at least slightly confident that they could start walking on their own or with at least another person without a group leader.

## DISCUSSION

Our findings from this study suggest that the culturally adapted WWE programme is acceptable and relevant to a UK population. Recruiting physically inactive adults with arthritis and MSK conditions through general practice was successful. Once enrolled, the majority of participants engaged with and successfully completed the programme, even maintaining their physical activity levels after the programme had concluded. Participants reported high levels of satisfaction with WWE overall and stated they would recommend it to family or friends. The findings provide a signal that this intervention is effective and is feasible to implement community-wide within the UK. A majority of the measures show improvement over six weeks, with this improvement being maintained to some extent at 18 weeks, for example, pain symptoms, HRQOL (EQ-5D-5L) and life satisfaction. A larger sample size would be needed to assess whether different mode of delivery have differential effects.

Various issues with and challenges to culturally adapting evidence-based interventions have previously been described in the literature [38, 39]. The particularities of culture, geography, and health care systems that characterize specific places can make translating elements of intervention to new places difficult. To-date, only a small number of evidence-based physical activity interventions for individuals with arthritis/MSK conditions have been successfully adapted to populations from those for whom they were originally developed. “Fit & Strong!” is an example of a CDC-recommended community-based physical activity programme combining aerobic walking, strength and flexibility training and health education to optimize outcomes among individuals with OA [40, 41]. This programme has been culturally adapted to and successfully implemented in Portugal [42]. Another example is the “Good Life with osteoarthritis in Denmark” (GLA:D) programme which was designed to improve uptake of evidence-based guidelines in the clinical care of knee and hip OA. This programme has been introduced successfully in Canada and Australia with good outcomes [43, 44]. Although WWE has been successfully adapted for the Spanish-speaking population in the USA and has demonstrated benefit [45], we are the first study to demonstrate its acceptability and relevance outside the USA.

Despite the positive feedback, findings from the qualitative interviews with participants and key stakeholders indicated scope for developing additional supplementary WWE-UK materials. We received feedback about enhancing the accessibility of WWE-UK for individuals with lower levels of literacy, poorer eyesight, and for whom holding a book may cause difficulties due to their arthritis/MSK condition. Illiteracy has been demonstrated to be independently associated with poorer health in the general population [46], as well as among individuals with arthritis [47]. Poor eyesight, either due from older age or arthritis-related eye conditions such as dry eye, glaucoma, scleritis, or uveitis [48] may prohibit individuals from fully engaging with and benefiting from the WWE guidebook. In addition, many individuals experience loss of strength and dexterity in their hands due to age, OA or RA-related swollen finger joints [49, 50]. We have therefore developed an audio e-book to ensure that WWE-UK remains inclusive to anyone in the future who wishes to engage with this evidence-based programme, especially those with accessibility issues.

Previous research has highlighted that there are disparities in the types of individuals who volunteer to take part in research, as well as community-based health programmes such as WWE [51, 52]. Our strategy was to recruit inactive adults with arthritis and MSK conditions through general practice, as postal invitations is a demonstrated practical approach. We had a 10.3% response rate from a posted GP letter, with 6.7% of these individuals being eligible to take part in the study which is aligned with previous research [53]. The recruitment approaches may therefore be considered appropriate. However future research would benefit from examining how to improve reach into targeted community groups (e.g., younger, working-aged individuals, men, non-white British). This may include letters that are tailored to an individual or that more broadly target a specific demographic within the population [54]. Pleasingly we were also able to demonstrate an ability to recruit community members to be volunteer WWE group instructors. This provided evidence, in principle, that this intervention may be sustainable within a community setting. However, given the study was undertaken in one geographical location in Scotland, further exploration may be warranted to determine recruitment and sustainability in other localities, although there is no reason to think it would be different in other UK locations. However, further work would need to be done to adapt WWE to Low-to-Middle-Income Countries (LMIC) where walking may be viewed not as an optional leisure activity and extreme climate or social factors (e.g., gender-based norms) might present ­complex challenges to delivering this community-based activity programme.

### Strengths and limitations

To our knowledge, this is the first cultural adaptation of WWE outside of a North American context. Whilst numerous studies have demonstrated the effectiveness of this programme, no study to-date has undertaken such a detailed qualitative exploration of participant experience with WWE. While this project was undertaken in a single major UK city with a population of over 200,000 residents, we believe that our findings are generalizable to other areas of Scotland and the UK more widely. There is scope for further exploration of WWE-UK in remote and rural areas, particularly among island-dwelling adults living with arthritis/MSK conditions. However, we have demonstrated that WWE is feasible and acceptable in a UK context and positively impacts on the physical health and well-being of participants, mirroring findings from the USA [22, 45, 51].

This study is not without limitations. Our study participants were predominantly White British, and future research among Black, Asian and Minority Ethnic (BAME) communities would be warranted to better understand relevance and acceptability in these populations [55]. Although 92% (48.8 million) hold English as their main language in England and Wales according to the 2011 Census, there may be a need to culturally adapt and translate WWE-UK into other languages predominately spoken by those not proficient in English (i.e., Polish, Punjabi, Urdu, Bengali, Gujarati, or Arabic) [56]. While our study attracted more men to WWE than previously reported (~30% vs. 15–25%) [22, 45, 51], more work should be done to understand how best to use WWE for men’s health promotion [57, 58] and reducing loneliness and social isolation in men [59]. We also recruited individuals to the study over the summer holidays. While this allowed us to take advantage of the milder weather and longer days due to the season, it may also have discouraged individuals with other commitments expressing an interest in the research if they were not available to take part. Although we offered a range of times and days of the walks, some individuals may have had schedule conflicts or not found a particular walk route convenient due to its location and chosen not to take part in the study. These types of logistics issues are not unique to this study as they are also common for community-based programmes.

## CONCLUSION

This study has demonstrated the acceptability and relevance of WWE, an evidence-based walking programme for adults with arthritis and MSK conditions designed in the USA, to a UK population. We have built on and optimized WWE materials to ensure inclusivity and accessibility. In addition to clinicians and health care providers, other commissioners of physical activity—particularly those responsible for older adults, should consider incorporating WWE-UK into programmatic offerings and also as part of social prescribing and physical activity referral schemes. WWE-UK would support key policy initiatives such as Scotland’s National Physical Activity Pathway and the National Walking Strategy, particularly post COVID-19, as a way to promote physical activity and reduce social isolation and loneliness in this at-risk ­population [60]. It also aligns with the Ageing Grand Challenge, recently launched by the UK Government to promote healthier, longer, and more connected lives by 2035 through innovation and dedicated funding. This study suggests WWE holds great promise for wider uptake outside the USA.

## Supplementary Material

Supplementary material is available at *Translational Behavioral Medicine* online.

E-mail: kevin.stelfox@abdn.ac.uk (KS), g.j.macfarlane@abdn.ac.uk (GJM), p.mcnamee@abdn.ac.uk (PM), z.morrison1@rgu.ac.uk (ZM), Toby.Smith@uea.ac.uk (TOS)

ibad032_suppl_Supplementary_Material

## WALK WITH EASE RESEARCH STUDY TEAM (IN ADDITION TO AUTHORS)

Aileen Neilson (Research Fellow), Santosh Gaihre (Research Fellow), Rosalind Rae (Research Fellow), Sarah Murdoch (Study Coordinator), Zoe Henderson (Study Coordinator), and Michelle Cloude (Project Assistant).

## Notes

**Funding:** This study was funded as a joint working programme between Versus Arthritis (previously Arthritis Research UK, Chesterfield, UK) and Pfizer Ltd, in partnership with the Chief Scientist’s Office of the Scottish Government [grant number 21414]. They had no role in the collection, analysis and interpretation of data or in the writing of the report.
