# Supplementary material for: Bringing the Walk with Ease Programme to the UK: a mixed-methods study to assess the relevance, acceptability, and feasibility of implementation for people with arthritis and musculoskeletal conditions
Source: Transl Behav Med. 2023 Jun 15;13(11):851–66. doi: 10.1093/tbm/ibad032 (PMC10631876; doi:10.1093/tbm/ibad032)
Supplement: ibad032_suppl_Supplementary_Material [file ibad032_suppl_supplementary_material.docx]

**Supplementary Material File 1: Participant Information and Physical Performance Assessment**

|  | **Source** | **Items** |  | **Assessment [weeks]** | | | | |
| --- | --- | --- | --- | --- | --- | --- | --- | --- |
|  |  |  |  | **0** | **2** | **4** | **6** | **18** |
| **Demographic data** |  |  |  |  |  |  |  |  |
| Age (Date of Birth) | Q | 1 |  | ✓ |  |  |  |  |
| Gender | Q | 1 |  | ✓ |  |  |  |  |
| Present Marital Status | Q | 1 |  | ✓ |  |  |  | ✓ |
| Ethnicity | Q | 1 |  | ✓ |  |  |  |  |
| Current Occupation | Q | 1 |  | ✓ |  |  | ✓ | ✓ |
| Current Employment Status | Q | 1 |  | ✓ |  |  | ✓ | ✓ |
| Unpaid or Voluntary Work | Q | 2 |  | ✓ |  |  |  | ✓ |
| Weekly Net Household Income | Q | 1 |  | ✓ |  |  | ✓ | ✓ |
| Educational Attainment | Q | 1 |  | ✓ |  |  |  |  |
| Current Smoking Status | Q | 1 |  | ✓ |  |  | ✓ | ✓ |
| Household Characteristics | Q | 2 |  | ✓ |  |  |  |  |
| Characteristics of study population |  |  |  |  |  |  |  |  |
| Overall health (SF-12) | Q | 1 |  | ✓ |  |  |  |  |
| Physical activity (self-reported) | Q | 1 |  | ✓ |  |  |  |  |
| Experience of fatigue for more than 3 months | Q | 1 |  | ✓ |  |  |  |  |
| Type of Arthritis or MSK Condition | Q | 1 |  | ✓ |  |  |  |  |
| Disease duration (years) | Q | 1 |  | ✓ |  |  |  |  |
| Presence of other co-morbidities | Q | 1 |  | ✓ |  |  |  |  |
| Previous and current pharmacological therapies | Q | 1 |  | ✓ |  |  |  |  |
| Height and Weight (BMI) | P | N/A |  | ✓ |  |  | ✓ | ✓ |
| Hip to Waist Ratio | P | N/A |  | ✓ |  |  | ✓ | ✓ |
| **Primary Outcomes** |  |  |  |  |  |  |  |  |
| Lower extremity strength (timed chair stands) | P | N/A |  | ✓ |  |  | ✓ |  |
| Turning ability and balance (Tinetti Performance-Mobility Assessment tool) | P | N/A |  | ✓ |  |  | ✓ |  |
| Balance (single-leg stance) | P | N/A |  | ✓ |  |  | ✓ |  |
| Functional mobility (six-foot walking speed test) | P | N/A |  | ✓ |  |  | ✓ |  |
| Aerobic endurance (two-minute step test) | P | N/A |  | ✓ |  |  | ✓ |  |
| Physical function (PROMIS PF 20) | Q | 20 |  | ✓ |  |  | ✓ | ✓ |
| Arthritis symptoms (VAS pain, stiffness and fatigue) | Q | 3 |  | ✓ |  |  | ✓ | ✓ |
| **Secondary Outcomes** |  |  |  |  |  |  |  |  |
| Arthritis Self-Efficacy | Q | 11 |  | ✓ |  |  | ✓ | ✓ |
| Rheumatology Attitudes Index (RAI) | Q | 15 |  | ✓ |  |  | ✓ | ✓ |
| Self-Efficacy for Physical Activity (SEPA) | Q | 5 |  | ✓ |  |  | ✓ | ✓ |
| Health-related quality of life (EQ-5D-5L) | Q | 5 |  | ✓ |  |  | ✓ | ✓ |
| Clinical Global Impression of Change | Q | 1 |  |  |  |  | ✓ | ✓ |
| Global Life Satisfaction | Q | 1 |  | ✓ |  |  | ✓ | ✓ |
| Physical Activity (EPAQ2) | Q | 77 |  | ✓ |  |  | ✓ | ✓ |
| Well-being (ICECAP) | Q | 5 |  | ✓ |  |  | ✓ | ✓ |
| Outcome Expectations for Exercise | Q | 2 |  | ✓ |  |  |  |  |
| Feelings and Behaviour (Social Desirability) | Q | 10 |  | ✓ |  |  |  |  |
| **Quantitative evaluation** |  |  |  |  |  |  |  |  |
| Patient preference for group allocation (randomisation preference questionnaire) | Q | 1 |  | ✓ |  |  |  |  |
| Patient adherence to the Walk With Ease Programme (attendance records) | R | 1 |  |  |  |  | ✓ |  |
| Patient acceptability (Walk With Ease Programme Evaluation) | Q | 10 |  |  |  |  | ✓ | ✓ |
| **Qualitative process evaluation** |  |  |  |  |  |  |  |  |
| Participant observation (ethnography) | O | N/A |  |  | ✓ | ✓ | ✓ |  |
| Biographical narrative interviews | I | N/A |  |  |  |  | ✓ |  |
| Semi-structured interviews with community partners | I | N/A |  |  |  |  | ✓ |  |
| **Economic evaluation** |  |  |  |  |  |  |  |  |
| Care Received from GP Surgery | Q | 1 |  |  |  |  | ✓ | ✓ |
| Prescribed Medication/s | Q | 2 |  |  |  |  | ✓ | ✓ |
| Non-Prescribed Medication/s | Q | 2 |  |  |  |  | ✓ | ✓ |
| Complementary Therapy Treatment | Q | 1 |  |  |  |  | ✓ | ✓ |
| Cost associated with delivery of the programme | E | N/A |  |  |  |  | ✓ | ✓ |
| EQ5D5L |  |  |  |  |  |  |  |  |

*Key: Q, data derived from questionnaires (self-report); I, interviews performed; S, information obtained during pre-study invite; P, information obtained during physical assessments, O, participant observation; R, attendance record kept by walk leader; E, economic evaluation conducted by research team.*

**Description of instruments used and reported on in Table 2 of this manuscript**

Physical Performance Measures

- Lower extremity strength, assessed with timed chair stands (five times sit to stand test). This test has demonstrated excellent reliability for community-dwelling elders [1,2] and those with low back pain [3] and OA [4] and validity in community-dwelling elderly [5,6], low back pain [3] and RA [7];
- Turning ability and balance, assessed using a timed 360^◦^ turn test within the Tinetti Performance-Mobility Assessment tool [8]. This is a reliable and valid measure of balance capability [9];
- Balance will be additionally assessed with the single-leg stance, as described by Lord et al [10], which has previously demonstrated reliability and validity compared to other measures of balance in community-dwelling individuals.
- Functional mobility, assessed with a six-foot walking speed test which has demonstrated reliability and validity [11,12];
- Aerobic endurance, assessed with a two-minute step test which has demonstrated reliability for older adults [13].

Arthritis Symptoms

- Three measures of arthritis symptoms (pain, stiffness and fatigue) will be included in the self-report survey using visual numeric rating scale (0-10) to rate symptom severity in the past seven days (i.e., pain, stiffness, and fatigue) [14,15].

Psychosocial Impact

- ICEpop CAPability Instrument (ICECAP-A) is a self-report instrument that records levels of capability and has a focus on wellbeing, which is measured with five questions across five attributes – attachment, stability, achievement, enjoyment, and autonomy. [16];
- EQ-5D-5L is a self-report measure of health-related quality of life covering five dimensions: mobility, self-care, usual activities, pain/discomfort, and anxiety/depression [17];
- Global Life Satisfaction. Life satisfaction will be assessed at baseline, six-weeks and 18 weeks by asking participants to rate from 1 (“completely dissatisfied”) to 7 (“completely satisfied”) the extent to which they are dissatisfied or satisfied with their life overall [18];
- Arthritis Self-Efficacy. Confidence for managing arthritis will be assessed using this 11-item instrument, which has two subscales: pain (5 items); and symptoms (6 items). Each item is scored from 1–10 and averaged over the subscales. Higher scores indicate arthritis self-efficacy [19];
- Rheumatology Attitudes Index (RAI). This is a 15-item scale assessing rheumatology attitudes. Perceived helplessness is measured with the 5-item helplessness subscale of the RAI. Each item is scored from 1–5 and averaged to give a total helplessness score. A higher score indicates greater helplessness [20];
- Self-Efficacy for Physical Activity (SEPA). Confidence to engage in PA despite barriers will be assessed using this 5-item scale, where each item is scored from 1–5 and averaged to create a total score. A higher score indicates greater levels of PA self-efficacy [21];
- Outcome Expectations for Exercise. This 9-item scale measures outcome expectations for exercise specifically for the older adult, where respondents are asked to strongly agree (1) to strongly disagree (5) [22].

**References – Supplementary Material File 1**

1. Bohannon, R. W., Shove, M. E., Barreca, S. R., Masters, L. M., & Sigouin, C. S. (2007). Five-repetition sit-to-stand test performance by community-dwelling adults: A preliminary investigation of times, determinants, and relationship with self-reported physical performance. *Isokinetics Exerc Sci*;*15*(2):77-81.
2. Tiedemann, A., Shimada, H., Sherrington, C., Murray, S., & Lord, S. (2008) The comparative ability of eight functional mobility tests for predicting falls in community-dwelling older people. *Age Ageing Jul;37*(4):430-435.
3. Simmonds, M. J., Olson, S. L., Jones, S., Hussein, T., Lee, C. E., Novy, D., et al. (1998). Psychometric characteristics and clinical usefulness of physical performance tests in patients with low back pain. *Spine* (Phila Pa 1976) Nov 15;23(22):2412-2421.
4. Lin, Y., Davey, R., & Cochrane, T. (2001) Tests for physical function of the elderly with knee and hip osteoarthritis. *Scand J Med Sci Sports* *11*(5):280-286.
5. Lord, S. R., Murray, S. M., Chapman, K., Munro, B., & Tiedemann, A. (2002). Sit-to-stand performance depends on sensation, speed, balance, and psychological status in addition to strength in older people*. J Gerontol A Biol Sci Med Sci Aug;57*(8):M539-43.
6. Schaubert, K. L. & Bohannon, R. W. (2005). Reliability and validity of three strength measures obtained from community-dwelling elderly persons. *J Strength Cond Res* *Aug;19*(3):717-720.
7. Newcomer, K. L., Krug, H. E., Mahowald, M. L. (1993) Validity and reliability of the timed-stands test for patients with rheumatoid arthritis and other chronic diseases. *J Rheumatol;20*(1):21-27.
8. Tinetti, M. E. & Ginter, S. F. (1988). Identifying mobility dysfunctions in elderly patients. Standard neuromuscular examination or direct assessment? *JAMA Feb 26*;259(8):1190-1193.
9. Whitney, S. L., Poole, J. L., & Cass, S. P. (1998). A review of balance instruments for older adults. *American Journal of Occupational Therapy 52*(8):666-671.
10. Lord, S. R., Clark, R. D., & Webster, I. W. (1991). Postural stability and associated physiological factors in a population of aged persons. J *Gerontol 46*(3):M69-76.
11. Wolf, S.L., Catlin, P.A., Gage, K., Gurucharri, K., Robertson, R., & Stephen, K. (1999). Establishing the reliability and validity of measurements of walking time using the Emory Functional Ambulation Profile. *Phys Ther 79*(12):1122-1133.
12. Jones, C. J. & Rikli, R. E. (2016). Measuring functional fitness of older adults. *Int J Active Aging 1*:25-30.
13. Fries, J. F., Krishnan, E., Rose, M., Lingala, B., & Bruce. B. (2011). Improved responsiveness and reduced sample size requirements of PROMIS physical function scales with item response theory. *Arthritis Res Ther 13*(5):R147.
14. Lorig, K., Stewart, A., Ritter, P., Gonzalez, V., Laurent, D., & Lynch, J. (1996). Outcome measures for health education and other health care interventions. Thousand Oaks (CA): *Sage Publications*.
15. Ritter, P.L., Gonzalez, V. M., Laurent, D.D., & Lorig, K. R. (2006). Measurement of pain using the visual numeric scale. *J Rheum 33*(3):574-580.
16. Al-Janabi, H., Flynn, T. N., & Coast, J. (2012). Development of a self-report measure of capability wellbeing for adults: the ICECAP-A. *Qual Life Res 21*(1):167-176.
17. Herdman, M., Gudex, C., Lloyd, A., Janssen, M., Kind, P., Parkin, D., et al. (2011). Development and preliminary testing of the new five-level version of EQ-5D (EQ-5D-5L). *Qual Life Res 20*(10):1727-1736.
18. Waldron S. (2010) Measuring subjective wellbeing in the UK. Newport: *Office for National Statistics*.
19. Lorig, K., Chastain, R.L., Ung, E., Shoor, S., & Holman, H.R. (1989). Development and evaluation of a scale to measure perceived self-efficacy in people with arthritis. *Arthritis Rheum 32*(1):37-44.
20. DeVellis, R. F. & Callahan, L.F. (1993). A brief measure of helplessness in rheumatic disease: the helplessness subscale of the Rheumatology Attitudes Index*. J Rheumatol 20*(5):866-869.
21. Marcus, B. H., Selby, V. C., Niaura, R. S., & Rossi, J.S. (1992). Self-efficacy and the stages of exercise behavior change. *Res Q Exerc Sport 63*(1):60-66.
22. Resnick, B., Zimmerman, S.I., Orwig, D., Furstenberg, A-L., & Magaziner, J. (2000). Outcome expectations for exercise scale: Utility and psychometrics. *J Geron: Soc Sci 55(*6):S352-S356.

**Supplementary Material File 2: Timeline**

**
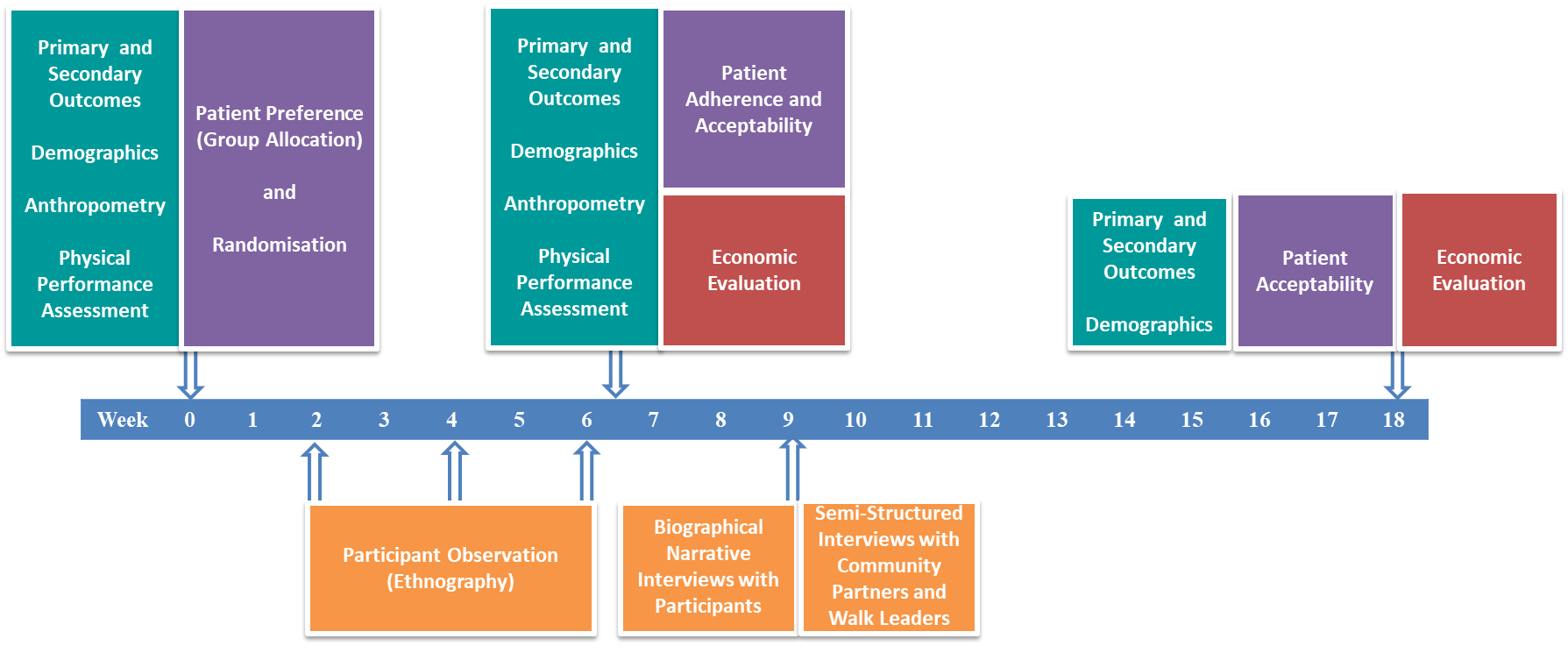
**

**Supplementary Material File 3: Biographical Narrative Interview Topic Guide**

**Primary narrative prompts:**

- *Background information:* Reaffirming study aims, confidentiality, use of data, voluntary participation etc.
- Initial introductory question: name, age, sex, condition, identification/characteristics of the participant including type of arthritis or MSK condition.
- *Initial narrative question:* can you tell me please what life is like living with your Arthritis or MSK condition?

**Secondary narrative question prompts (if not covered in the main narrative phase):**

*Participant’s condition/quality of life/self-management*

- So in terms of living with your condition, what has been the impact on your life in terms of what you did before and can’t do now/or find difficult doing now?
- Did your condition impact upon your ability to be physically active? What barriers and facilitators did/have you experienced in relation to being physically active?
- Any other challenges related to your condition?
- How well do you cope with your condition and the challenges you have mentioned/do you cope?
- What do you do to manage this?
- In terms of where you are with your condition, what would like to do, that you are not able to do at the moment?
- Thinking ahead, what do you think would a make a difference to you to improve your health and ability to manage your condition further?

*Walk With Ease Programme Impact*

- How do you feel generally about the Walk With Ease (WWE) programme?
- How closely have you followed the WWE programme guidelines (verbal confirmation and visual?)

Do you think the WWE programme has had an impact on you / your condition such as being physically active, impacting your symptoms, your mood and/or your quality of life?

If yes – please could you tell me in what way it has had an impact on you?

If no – please could you explain why you feel it hasn’t had an impact on you?

- What impact has the WWE programme had on you (if any)? And with what outcomes?
- What have been the benefits and challenges of the WWE programme for you?
- Do you feel that WWE has impacted upon your general health and/or your arthritis or MSK condition in any way? How? If not, why?
- Do you think that your perception of being physically active whilst living with your condition has changed over the course of the WWE study? Why/why not?

Do you think that there is any way in which we can improve the WWE programme?

If yes – please could you give me some examples of how we might improve the WWE in the future?

If no – please could you explain why?

- Over the longer term would you use the WWE programme, or a similar programme?

If yes - please could you tell me why you would use the WWE programme / other programme? What barriers and facilitators do you think you might encounter in engaging in this type of programme over the longer term?

If no – please could you explain why?
